# Smoking status and subsequent gastric cancer risk in men compared with women: a meta-analysis of prospective observational studies

**DOI:** 10.1186/s12885-019-5601-9

**Published:** 2019-04-24

**Authors:** Wen-Ya Li, Yunan Han, Hui-Mian Xu, Zhen-Ning Wang, Ying-Ying Xu, Yong-Xi Song, Hao Xu, Song-Cheng Yin, Xing-Yu Liu, Zhi-Feng Miao

**Affiliations:** 1 Department of Thoracic surgery, First Hospital of China Medical University, Shenyang, Liaoning Province China; 2 Department of Breast Surgery, First Hospital of China Medical University, Shenyang, Liaoning Province China; 3 Division of Public Health Sciences, Department of Surgery, Washington University School of Medicine, Saint Louis, MO USA; 4 Department of Surgical Oncology, First Hospital of China Medical University, Shenyang, Liaoning Province China; 5 Department of Medical Oncology, Shengjing Hospital of China Medical University, Shenyang, Liaoning Province China

**Keywords:** Smoking, Gastric cancer, Cancer risk, Sex, Meta-analysis

## Abstract

**Background:**

Smoking is one of the well-established risk factors for gastric cancer incidence, yet whether men are more or equally susceptible to gastric cancer due to smoking compared with women is a matter of controversy. The aim of this study was to investigate and compare the effect of sex on gastric cancer risk associated with smoking.

**Methods:**

We conducted a systemic literature search in MEDLINE, EMBASE, and the Cochrane CENTRAL databases to identify studies published from inception to December 2018. We included prospective observational studies which reported effect estimates with 95% confidence intervals (CIs) for associations of current or former smokers with the incidence of gastric cancer by sex. We calculated the ratio of relative risk (RRR) with corresponding 95% CI based on sex-specific effect estimates for current or former smokers versus non-smokers on the risk of gastric cancer.

**Results:**

We included 10 prospective studies with 3,381,345 participants in our analysis. Overall, the summary RRR (male to female) for gastric cancer risk in current smokers was significantly increased compared with non-smokers (RRR: 1.30; 95% CI: 1.05–1.63; *P* = 0.019). Furthermore, there was no significant sex difference for the association between former smokers and gastric cancer risk (RRR: 1.20; 95% CI: 0.92–1.55; *P* = 0.178). However, the result of sensitivity analysis indicated the pooled result was not stable, which was altered by excluding a nested case-control study (RRR: 1.31; 95% CI: 1.10–1.57; *P* = 0.002).

**Conclusion:**

This systematic review showed a potential sex difference association between current smokers and the risk of gastric cancer. The sex differential in smokers can give important clues for the etiology of gastric cancers and should be examined in further studies.

**Electronic supplementary material:**

The online version of this article (10.1186/s12885-019-5601-9) contains supplementary material, which is available to authorized users.

## Background

Gastric cancer is the fifth most common cancer and the third leading cause of cancer mortality worldwide, despite its decreasing incidence in recent decades [1, 2]. Moreover, there is a sex-specific disparity in gastric cancer incidence. Incidence rates are 2-fold higher in men than in women worldwide [1]. Gastric cancer is a multifactorial disease, and both environmental and genetic factors have a role in its etiology. Common risk factors include older age, *Helicobacter pylori* infection, coffee, dairy products, red meat consumption, tobacco smoking, radiation, high body mass index, and family history [3–8]. There are also geographic, ethnic, and sex differences in the incidence of gastric cancer.

Previous studies have indicated that environmental factors could affect gastric cancer risk more prominently than genetic factors [9–11]. Several studies have suggested that smoking was associated with a higher risk of gastric cancer and a previous meta-analysis considered smoking to be most important behavioral risk factor for gastric cancer [12]. However, the role of sex differences remain controversial. Clarifying the association of smoking status with the risk of gastric cancer in men compared with women is particularly important since the prevalence of smoking in women is increasing and now tobacco use is seen as a “contemporary epidemic” in women in the United States and many other countries. A potential sex difference could help identify high-risk population groups for gastric cancer in smokers, allowing for the formulation of effective primary prevention strategies. Therefore, we performed a large-scale examination of the available prospective observational studies to explore the association between smoking status and gastric cancer risk by sex. We further evaluated the sex difference according to the baseline characteristics of the participants.

## Methods

### Data sources, search strategy, and selection criteria

This systematic review was conducted and reported according to the Preferred Reporting Items for Systematic Reviews and Meta-Analysis (PRISMA) Statement issued in 2009 [13]. Relevant articles were systematically searched in MEDLINE, EMBASE, and the Cochrane CENTRAL electronic databases from database inception to December 2018. We included studies that investigated humans without language restrictions and regardless of publication status (published, in the press, or in progress). The studies reporting associations between smoking status and gastric cancer risk were searched using strategies of a combined text and medical subjects headings (MeSH): (“smoke” OR “smoking” OR “nicotine” OR “tobacco” OR “lifestyle” OR “lifestyles” OR “cigarette”) AND (“gastric” OR “stomach” OR “cardia”) AND (“cancer” OR “tumor” OR “neoplasm”) AND (“nested case control” OR “cohort” OR “prospective”). Furthermore, we also manually checked the reference lists of identified reports for other potentially relevant studies. If the same population was reported more than once, the most comprehensive and recently published article was used. The study topic, study design, exposure, population, and reported outcomes were used to identify relevant studies.

Two authors independently performed a literature search and study selection, and disagreements between two authors were settled by a discussion in a group until a consensus was reached. A study was deemed eligible if it met the following inclusion criteria: (1) the study design was a prospective observational study; (2) the study evaluated the association of smoking status with gastric cancer risk; and (3) the associations between smoking status and gastric cancer risk in men and women were both reported.

### Data collection and quality assessment

Two authors independently collected and extracted data from the included studies, and disagreements were resolved by a group discussion. The data collected from the included studies contained the following items: first author, publication year, country, sex, sample size of men and women, mean age for men and women, number of participants who had never smoked (non-smokers) for men and women, number of former smokers in men and women, number of current smokers in men and women, follow-up duration, reported outcomes, and adjusted factors.

We assessed the methodological quality of the included studies using the Newcastle-Ottawa Scale (NOS) [14], which has been partially validated for evaluating the quality of observational studies included in meta-analyses. The NOS is based on selection (4 items), comparability (1 item), and outcome (3 items), and provides a “star system” range of 0–9 to evaluate study quality. Two authors independently performed quality assessments and disagreements were settled by a group discussion.

### Statistical analysis

The associations between smoking status and gastric cancer risk in men and women were determined based on the relative risk (RR), hazard ratio (HR), or odds ratio (OR), and the 95% confidence intervals (CIs) in each individual study. HR is considered equivalent to RR in prospective observational studies, and OR could also be assumed to be equivalent to the RR due to the low incidence of gastric cancer. We calculated the ratio of RRs (RRR) for current or former smokers versus non-smokers and the risk of gastric cancer based on sex-specific RRs in individual studies [15]. We used random-effects models to calculate the summary RRR and compared the sex differences in gastric cancer risk in current smokers, former smokers, or non-smokers [16, 17].

Heterogeneity among studies was shown by the I^2^ and Q statistics, and *P* values < 0.10 mean significant heterogeneity [18, 19]. A sensitivity analysis was performed by systematically excluding each study individually to evaluate its influence on the meta-analysis [20]. The potential sources of heterogeneity in estimates of the impact of current and former smokers based on follow-up duration were explored by using univariate meta-regression [21]. Subgroup analyses for the sex differences in the association between smoking status and gastric cancer risk were based on publication year, country, follow-up duration, reported outcomes, whether or not the studies adjusted for BMI or alcohol consumption, and study quality. Publication bias was explored visually using funnel plots and statistically using Egger’s and Begg’s tests [22, 23]. All *P* values were two-sided with significance defined as *P* < 0.05. Statistical analyses were conducted using STATA software (version 10.0; Stata Corporation, College Station, TX, USA).

## Results

### Literature search

**Fig. 1:**
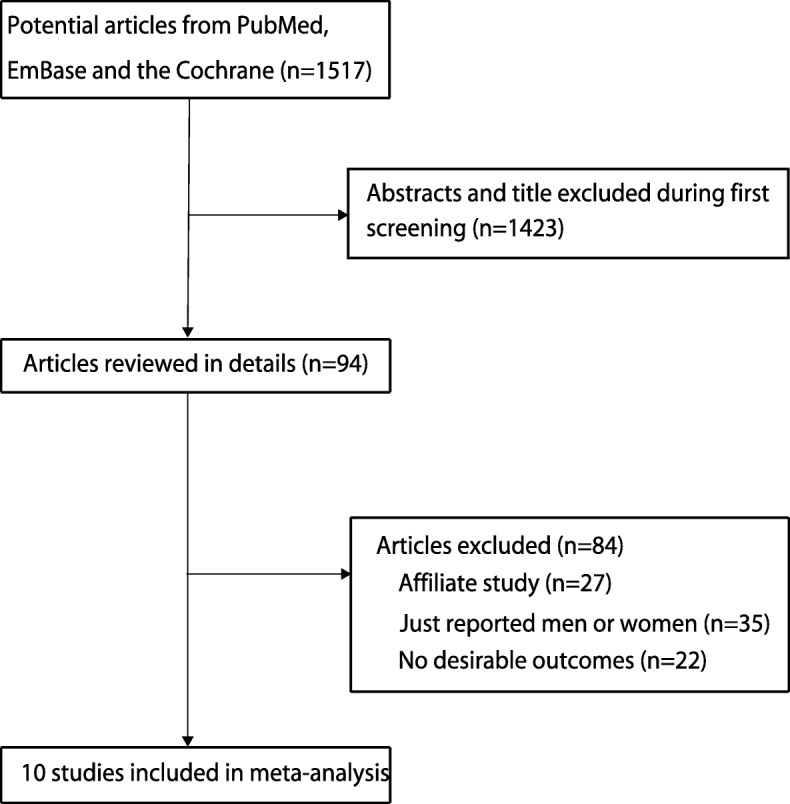
Flow diagram of the literature search and trial selection process

A total of 1517 records from the initial search were identified, including 691 from MEDLINE, 757 from EMBASE, and 69 from the Cochrane CENTRAL. After discarding 1423 irrelevant or duplicate studies, 94 potential studies were selected for further reading. After detailed evaluating, 10 prospective observational studies were selected into the quantitative analysis [24–33]. The manual search of the reference lists of these studies did not yield any new eligible studies. The systematic review selection process is shown in Fig. 1, and the general characteristics of the included studies are displayed in Table 1.

**Table 1:** Baseline characteristic of studies included in the systematic review and meta-analysis

Study	Country	Sex	Sample size	Age (years)	Never smoker	Former smoker	Current smoker	Follow-up (years)	Reported outcomes	Adjusted factors	NOS score
JACC 2005 [24]	Japan	Men	43,482	57.4	9014	11,471	22,997	9.9	GC mortality	Age, smoking, alcohol intake, educational level, preference for salty foods and green-tea intake.	7
		Women	43,482	57.4	9014	11,471	22,997				
Kato 1992 [25]	Japan	Men	NA	> 30.0	NA	NA	NA	6.0	GC mortality	Age	6
		Women	NA	> 30.0	NA	NA	NA				
Tverdal 1993 [26]	Norway	Men	44,290	35.0–49.0	9334	10,467	18,400	13.3	GC mortality	Age, cholesterol, SBP, PI during leisure, BMI, height and number of cigarettes.	7
		Women	24,535	35.0–49.0	12,369	3079	8954				
Engeland 1996 [27]	Norway	Men	8905	23.0–57.0	NA	NA	NA	28.0	GC	NA	7
		Women	10,169	18.0–57.0	NA	NA	NA				
Lindblad 2005 [28]	UK	Men	6860	40.0–84.0	2956	678	1212	NA	GC	Age, calendar year, BMI, alcohol consumption and reflux	6
		Women	3335	40.0–84.0	1744	168	395				
Jee 2004 [29]	Korea	Men	830,139	45.0	166,858	190,932	473,179	8.0	GC mortality	Age	8
		Women	382,767	48.6	352,528	9569	20,669				
Chao 2002 [30]	US	Men	467,788	57.0	117,968	179,833	151,406	14.0	GC mortality	Age, race, education, family history of stomach cancer, consumption of high-fiber grain foods, vegetables, citrus fruits or juices, and use of vitamin C, multivitamins, and aspirin	8
		Women	588,053	56.0	326,835	122,455	122,465				
Akiba 1990 [31]	Japan	Men	122,261	> 40.0	NA	NA	NA	16.0	GC mortality	Prefecture of residence, occupation, attained age, and observation period	6
		Women	142,857	> 40.0	NA	NA	NA				
Gonzalez 2003 [32]	Europe (10 countries)	Men	148,182	51.6	49,678	36,791	36,643	5.0	GC	Age, sex, vegetables, fruits, processed meat, alcohol, BMI and educational level	7
		Women	322,046	51.6	191,037	41,887	58,319				
Nomura 2012 [33]	US	Men	82,683	60.1	25,466	42,251	14,966	7.3	GC	Age at cohort entry as a continuous variable, ethnicity as a strata variable, education, processed meat intake, BMI, alcohol intake, aspirin use, and family history of gastric cancer	8
		Women	99,758	59.6	56,463	28,930	14,365				

*GC* gastric cancer, *SBP* systolic blood pressure, *PI* physical activity, *BMI* body mass index

### Study characteristics

Ten studies with a total of 3,381,345 participants were included in our analysis. Among the studies, nine were prospective cohort studies [24–27, 29–33] and one was a nested case-control study [28]. The duration of follow-up for participants was 5.0–28.0 years, while 9753-1,212,906 individuals were included in each study. Three studies were conducted in Japan [24, 25, 31], one in Korea [30], two in Norway [26, 27], one in the UK [28], two in the US [30, 33], and one in 10 European countries [32]. The main study outcome in 6 studies was gastric cancer mortality, and the remaining 4 studies reported gastric cancer incidence. NOS scores were used to evaluate study quality [14], and a score ≥ 7 was regarded as high quality. Overall, three studies had scores of 8, four studies had scores of 7, and the remaining three studies had scores of 6.

### Sex differences for gastric Cancer risk in current smokers

**Fig. 2:**
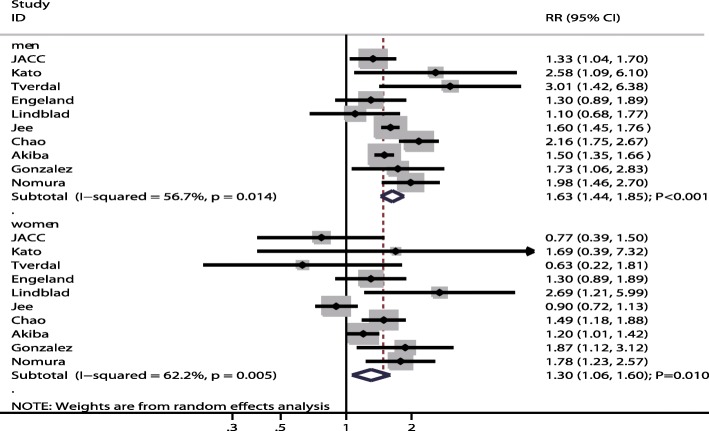
The associations of current smokers with the risk of gastric cancer in men and women separately

**Fig. 3:**
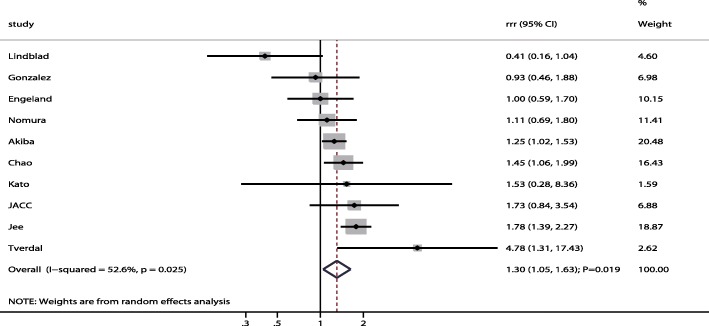
Sex difference of the association between current smokers and the risk of gastric cancer

All included studies reported sex differences in the association between gastric cancer risk and current smokers compared with non-smokers. We noted current smokers were associated with higher risk of gastric cancer when compared with non-smokers in men (RR: 1.63; 95% CI: 1.44–1.85; *P* < 0.001; Fig. 2) and women (RR: 1.30; 95% CI: 1.06–1.60; *P* = 0.010; Fig. 2). Further, the increased risk of gastric cancer in current smokers compared to non-smokers was higher in men than in women (RRR: 1.30; 95% CI: 1.05–1.63; *P* = 0.019; Fig. 3), with significant heterogeneity (I^2^ = 52.6%; *P* = 0.025). The result of the sensitivity analysis indicated that the sex differences in the association between current smokers and gastric cancer were affected by the exclusion of multiple studies due to the small numbers of cohorts included (Table 2). The results of the meta-regression analysis showed that follow-up duration was not a significant factor contributing to the sex differences of the association between current smokers and gastric cancer (Additional file 1). We used subgroup analyses to minimize heterogeneity among the included studies and evaluate the sex differences in subpopulations (Table 3). The summary RRR (male to female) for current smokers indicated an increased risk of gastric cancer in men when the study was conducted in Asia (RRR: 1.50; 95% CI: 1.17–1.91; *P* = 0.001), regardless of follow-up duration (follow-up duration ≥10.0 years [RRR: 1.33; 95% CI: 1.02–1.74; *P* = 0.037]; follow-up duration < 10.0 years [RRR: 1.46; 95% CI: 1.11–1.91; *P* = 0.006]), when the study reported gastric cancer mortality (RRR: 1.53; 95% CI: 1.24–1.89; *P* < 0.001), when the study did not adjust for BMI (RRR: 1.47; 95% CI: 1.24–1.74; *P* < 0.001), when the study did not adjust for alcohol consumption (RRR: 1.53; 95% CI: 1.20–1.94; P = 0.001), and when the study had a NOS score of 7 or 8 (RRR: 1.42; 95% CI: 1.11–1.81; *P* = 0.005).

**Table 2:** Sensitivity analysis for sex difference of gastric cancer (current smoker versus never smoker and former smoker versus never smoker)

Outcomes	Excluding study	RRR and 95% CI	*P* value	Heterogeneity (%)	*P* value for heterogeneity
Current smoker versus never smoker	JACC 2005	1.27 (1.01–1.61)	0.045	56.8	0.018
	Kato 1992	1.30 (1.03–1.63)	0.026	57.8	0.015
	Tverdal 1993	1.27 (1.04–1.57)	0.022	47.8	0.053
	Engeland 1996	1.34 (1.06–1.70)	0.015	54.5	0.024
	Lindblad 2005	1.38 (1.15–1.66)	0.001	35.7	0.132
	Jee 2004	1.22 (0.97–1.53)	0.089	38.1	0.114
	Chao 2002	1.27 (0.97–1.66)	0.080	57.4	0.016
	Akiba 1990	1.30 (0.98–1.73)	0.068	55.1	0.023
	Gonzalez 2003	1.34 (1.06–1.69)	0.014	55.0	0.023
	Nomura 2012	1.33 (1.04–1.70)	0.023	56.1	0.020
Former smoker versus never smoker	JACC 2005	1.20 (0.90–1.59)	0.220	48.4	0.060
	Kato 1992	1.20 (0.92–1.58)	0.175	46.9	0.068
	Tverdal 1993	1.20 (0.91–1.58)	0.187	47.7	0.063
	Engeland 1996	1.15 (0.87–1.53)	0.332	47.7	0.063
	Lindblad 2005	1.31 (1.10–1.57)	0.002	5.7	0.386
	Jee 2004	1.10 (0.78–1.56)	0.592	46.2	0.072
	Chao 2002	1.17 (0.83–1.64)	0.364	46.0	0.073
	Gonzalez 2003	1.26 (0.96–1.64)	0.095	40.8	0.107
	Nomura 2012	1.11 (0.85–1.44)	0.445	28.8	0.199

**Table 3:** Subgroup analysis for sex difference of gastric cancer (current smoker versus never smoker)

Group	RRR and 95% CI	*P* value	Heterogeneity (%)	P value for heterogeneity	*P* value for interaction test
Publication year					
2000 or after	1.27 (0.93–1.74)	0.130	60.6	0.027	0.921
Before 2000	1.32 (0.89–1.96)	0.172	38.2	0.183	
Country					
Asia	1.50 (1.17–1.91)	0.001	40.2	0.171	0.306
Europe or US	1.12 (0.77–1.64)	0.548	58.2	0.035	
Follow-up duration (years)					
10 or greater	1.33 (1.02–1.74)	0.037	44.7	0.143	0.573
< 10	1.46 (1.11–1.91)	0.006	22.4	0.272	
Outcomes					
GC incidence	0.92 (0.66–1.29)	0.628	16.5	0.309	0.023
GC mortality	1.53 (1.24–1.89)	< 0.001	39.3	0.143	
Adjusted BMI or not					
Yes	1.07 (0.53–2.15)	0.848	68.0	0.025	0.527
No	1.47 (1.24–1.74)	< 0.001	20.2	0.286	
Adjusted alcohol consumption					
Yes	0.99 (0.61–1.61)	0.971	49.6	0.114	0.331
No	1.53 (1.20–1.94)	0.001	50.2	0.090	
NOS score					
7 or 8	1.42 (1.11–1.81)	0.005	43.4	0.101	0.169
< 7	0.91 (0.41–2.02)	0.814	62.7	0.068	

### Sex differences in gastric Cancer risk for former smokers

**Fig. 4:**
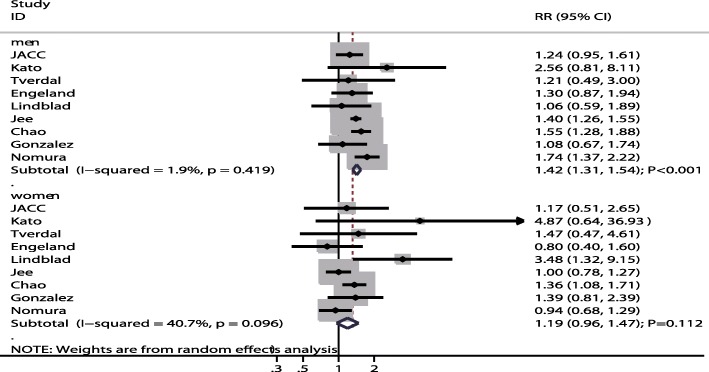
The associations of former smokers with the risk of gastric cancer in men and women separately

**Fig. 5:**
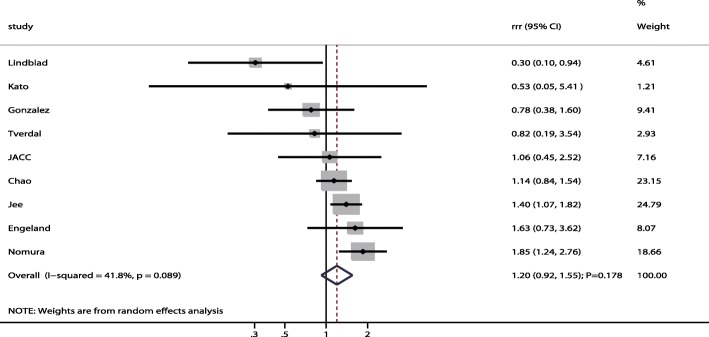
Sex difference of the association between former smoker and the risk of gastric cancer

A total of 9 studies reported sex differences in the relation between gastric cancer risk in former smokers compared to non-smokers. The summary result indicated former smokers were associated with an increased risk of gastric cancer in men (RR: 1.42; 95% CI: 1.31–1.54; P < 0.001; Fig. 4), while this association was not associated with statistically significant in women (RR: 1.19; 95% CI: 0.96–1.47; *P* = 0.112; Fig. 4). There was no significant difference for gastric cancer risk between former smokers and non-smokers in men compared with women (RRR: 1.20; 95% CI: 0.92–1.55; *P* = 0.178; Fig. 5), and potential significant heterogeneity was observed among the included studies (I^2^ = 41.8%; *P* = 0.089). Following the result of the sensitivity analysis, we excluded the study by Lindblad et al. [28], which used a nested case control design. After this exclusion, we could conclude that male former smokers had a significantly increased risk of gastric cancer over non-smokers compared to female former smokers (RRR: 1.31; 95% CI: 1.10–1.57; *P* = 0.002; Table 2). Meta-regression analysis indicated follow-up duration did not contribute a significant role with the sex difference of the relation between former smokers and gastric cancer (Additional file 1). Subgroup analyses indicated a higher risk of gastric cancer in male verses female former smokers when the study was conducted in Asia (RRR: 1.35; 95% CI: 1.05–1.74; *P* = 0.019; Table 4), follow-up duration < 10.0 years (RRR: 1.35; 95% CI: 1.02–1.80; *P* = 0.038), when the study reported gastric cancer mortality (RRR: 1.25; 95% CI: 1.03–1.51; *P* = 0.022; Table 4), when the study did not adjust for BMI (RRR: 1.26; 95% CI: 1.04–1.53; *P* = 0.019; Table 4), when the study did not adjust for alcohol consumption (RRR: 1.26; 95% CI: 1.04–1.53; *P* = 0.020; Table 4), and when the study had high study quality (RRR: 1.32; 95% CI: 1.09–1.59; *P* = 0.004; Table 4). Furthermore, male former smokers were associated with a lower risk of gastric cancer if the study had lower study quality (RRR: 0.34; 95% CI: 0.12–0.93; *P* = 0.036).

**Table 4:** Subgroup analysis for sex difference of gastric cancer (former smoker versus never smoker)

Group	RRR and 95% CI	*P* value	Heterogeneity (%)	*P* value for heterogeneity	*P* value for interaction test
Publication year					
2000 or after	1.17 (0.85–1.59)	0.332	60.0	0.029	0.731
Before 2000	1.28 (0.65–2.51)	0.471	0.0	0.534	
Country					
Asia	1.35 (1.05–1.74)	0.019	0.0	0.606	0.528
Europe or US	1.10 (0.73–1.66)	0.648	59.7	0.029	
Follow-up duration (years)					
10 or greater	1.17 (0.89–1.55)	0.252	0.0	0.638	0.417
< 10	1.35 (1.02–1.80)	0.038	27.3	0.240	
Outcomes					
GC incidence	1.03 (0.51–2.07)	0.936	73.9	0.009	0.436
GC mortality	1.25 (1.03–1.51)	0.022	0.0	0.726	
Adjusted BMI or not					
Yes	0.86 (0.38–1.93)	0.714	74.2	0.009	0.171
No	1.26 (1.04–1.53)	0.019	0.0	0.630	
Adjusted alcohol consumption					
Yes	0.92 (0.45–1.89)	0.828	73.9	0.009	0.213
No	1.26 (1.04–1.53)	0.020	0.0	0.592	
NOS score					
7 or 8	1.32 (1.09–1.59)	0.004	12.1	0.337	0.001
< 7	0.34 (0.12–0.93)	0.036	0.0	0.680	

### Publication Bias

**Fig. 6:**
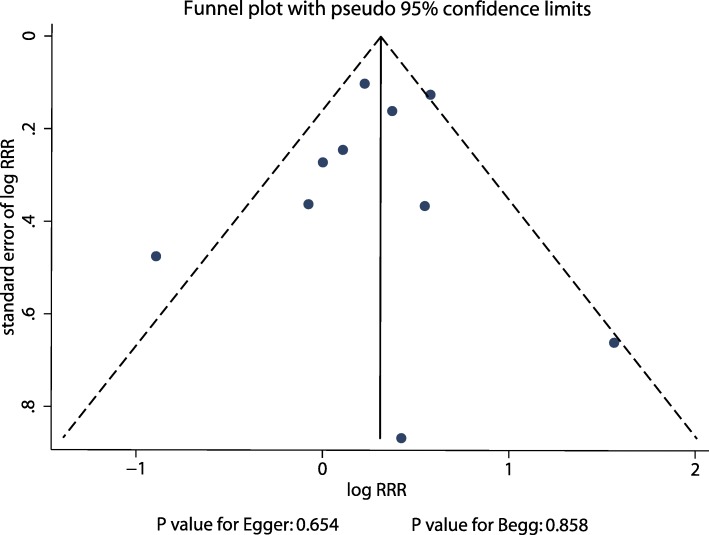
Funnel plots for current smokers versus non-smokers

**Fig. 7:**
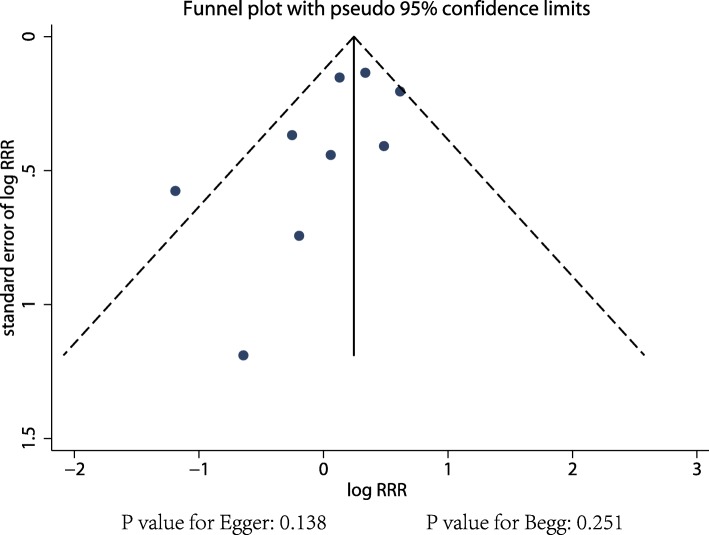
Funnel plots for former smokers versus non-smokers

Reviewing the funnel plots could not rule out the potential publication bias contributing to the sex differences in gastric cancer risk. The Egger’s and Begg’s test results showed no evidence of publication bias for sex differences in the association between current smokers and gastric cancer risk (Fig. 6). Moreover, there was no significant publication bias for former smokers and gastric cancer risk (Fig. 7).

## Discussion

This analysis explored sex differences in the associations between smoking status and gastric cancer risk based on 10 prospective observational studies. In total, 3,381,345 participants from 9 prospective cohort studies and 1 nested case-control study were included with a broad range of characteristics. The results of our study suggested that male current smokers had a significantly higher risk of gastric cancer compared to women, while no sex differences were found for the association between former smokers and gastric cancer risk. Sensitivity and subgroup analyses might prove variable due to different baseline characteristics.

A previous study indicated that current smokers in men (RR: 1.62; 95% CI: 1.50–1.75) and women (RR: 1.20; 95% CI: 1.01–1.43) were associated with a significantly increased risk of gastric cancer when compared to non-smokers [12]. Furthermore, Tredaniel et al. indicated that the risk of gastric cancer among smokers was significantly increased compared with non-smokers, and the summary RR was higher in men than women (RR: 1.59 vs 1.11) [9]. Koizumi et al. pooled analyses of two prospective cohort studies in Japan and concluded that gastric cancer risk for current smokers (RR: 1.84; 95% CI: 1.39–2.43) and former smokers (RR: 1.77; 95% CI: 1.29–2.43) were increased compared to non-smokers [34]. Nishino et al. found that current smoking significantly increased the risk of gastric cancer in men (RR: 1.79; 95% CI: 1.51–2.12) and women (RR: 1.22; 95% CI: 1.07–1.38) [35]. The inherent limitations of those previous meta-analyses included the following: (1) although the risk of gastric cancer was different between men and women, the results from different studies might contribute important heterogeneity due to different populations; (2) the included participants were not reported with separate effect estimates in men and women, and there was no direct comparison in sex differences; (3) they did not calculate the associations between smoking status and gastric cancer risk in men and women; and (4) the study combined retrospective and prospective observational studies, which might introduce potential confounders. Therefore, we conducted a meta-analysis of prospective observational studies to evaluate sex differences in the relation between smoking status and gastric cancer risk.

The summary RRR indicated that male current smokers had a greater risk of gastric cancer than women. However, several studies included in our study reported inconsistent results. Lindblad et al. indicated that female current or former smokers were significantly associated with a higher risk of gastric cancer than non-smokers, while this association was not statistically significant in men [28]. The reason for this difference might be because this study was specifically designed as a nested case-control study, and there were imbalances in the number of participants in each smoking category. Gonzalez et al. indicated that current smokers associated with a higher risk of gastric cancer for both men and women, while there was no significant difference finding for gastric cancer risk between former smokers and non-smokers in men or women [32]. Engeland et al. indicated that current smokers or former smokers had no significant change in the risk for gastric cancer when compared with non-smokers for men or women [27]. Our analysis found that male current smokers had a significantly increased risk of gastric cancer, while no significant effect was shown in women [24, 25]. This higher risk of gastric cancer in male smokers than in female smokers might be attributed to a fewer number of cigarettes smoked and shorter smoking duration for women than men. In addition, the risk of gastric cancer due to smoking was higher in men than women, which might affect the sex difference of smoking status and subsequent risk of gastric cancer [36]. Finally, the high rate of alcohol consumption in men was significantly associated with the prevalence of smoking, especially for alcoholism, which was associated with an increased risk of gastric cancer [37].

The findings of the subgroup analyses indicated that the sex differences in gastric cancer risk for current smokers might be affected by country, reported outcomes, whether BMI or alcohol were adjusted for, and study quality. Male former smokers were associated with a higher risk of gastric cancer than female former smokers when the study was conducted in Asia, the outcome was gastric cancer mortality, the study did not adjust for BMI or alcohol consumption, and the study had high study quality. However, female former smokers were associated with a higher risk of gastric cancer than men when the included studies had lower study quality. One possible reason for the locational difference could be that different types of tobacco available between Asian and Western countries, which could have different effects on gastric cancer risk. Furthermore, men might smoke more cigarettes and have a longer duration of smoking than women, which might affect the gastric cancer mortality. Finally, the findings of the subgroup analyses may be variable due to the small cohorts included for several subsets. Therefore, a synthetic and comprehensive review was provided in this study.

We had three strengths in our study that should be highlighted. First, only prospective observational studies were included, which should eliminate the selection and recall biases inherent in retrospective observational studies. Second, the large sample size allowed us to quantitatively assess the association of smoking status and risk of gastric cancer, thus our findings are potentially more robust than the individual studies. Third, sex differences in the associations between smoking status and the risk of gastric cancer were directly compared among individual studies.

The limitations of our study were as follows: (1) the adjusted models were different in the included studies, and these factors might play essential roles in the development of gastric cancer; (2) the history of *Helicobacter pylori* infection is an important factor which is associated with a higher risk of gastric cancer, but none of the included studies adjusted for *Helicobacter pylori* and corresponding treatment strategies [3]; (3) the sex differences of the association between smoking status and gastric cancer risk were using dose-response meta-analytic approach, while cigarette smoke exposure as a continuous variable was not available in included studies; (4) although we did not find significant bias in our present work, publication bias was still an inevitable problem in a meta-analysis of published studies; and (5) the analysis used pooled data (individual data were not available) could not provide a more detailed relevant analysis and more comprehensive results.

## Conclusion

The results of this study suggested that current smoking might have a more important effect on gastric cancer risk in men than women, while no sex differences were found for the association between former smokers and gastric cancer risk. Furthermore, potential sex difference for the association between former smokers and gastric cancer risk was observed through sensitivity analysis. In addition, this significant sex-difference mainly focused on gastric cancer mortality, while no sex-difference of current or former smoking on gastric cancer incidence. Several factors might affect this sex difference in the risk of gastric cancer, and future studies should focus on other impact factors to analyze the sex difference of gastric cancer.

## Additional file

Additional file 1: **Supplemental 1.** Meta-regression analyses for the sex difference of the association between current or former smokers and gastric cancer based on follow-up duration. (DOC 6972 kb)

## Abbreviations

CIs: confidence intervals
HR: hazard ratio
MeSH: medical subjects headings
NOS: Newcastle-Ottawa Scale
OR: odds ratio
PRISMA: reporting items for systematic reviews and meta-Analysis
RR: relative risk
RRR: ratio of relative risk
