# Supplementary material for: Smoking status and subsequent gastric cancer risk in men compared with women: a meta-analysis of prospective observational studies
Source: BMC Cancer. 2019 Apr 24;19:377. doi: 10.1186/s12885-019-5601-9 (PMC6480657; doi:10.1186/s12885-019-5601-9)
Supplement: Supplementary file 1 — Supplemental 1. Meta-regression analyses for the sex difference of the association between current or former smokers and gastric cancer based on follow-up duration. (DOC 6972 kb) [file 12885_2019_5601_MOESM1_ESM.doc]

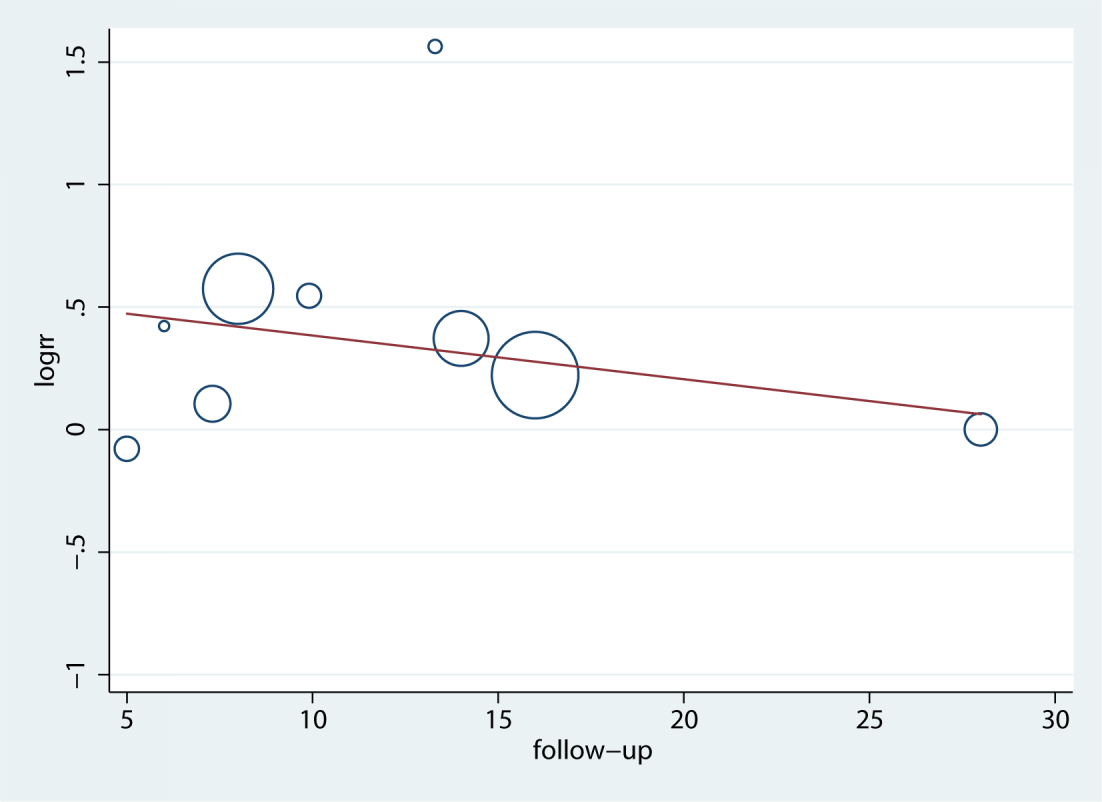


Figure S1. Meta-regression of sex differences in the relationship between current smokers and the risk of gastric cancer based on follow-up duration (P=0.271).


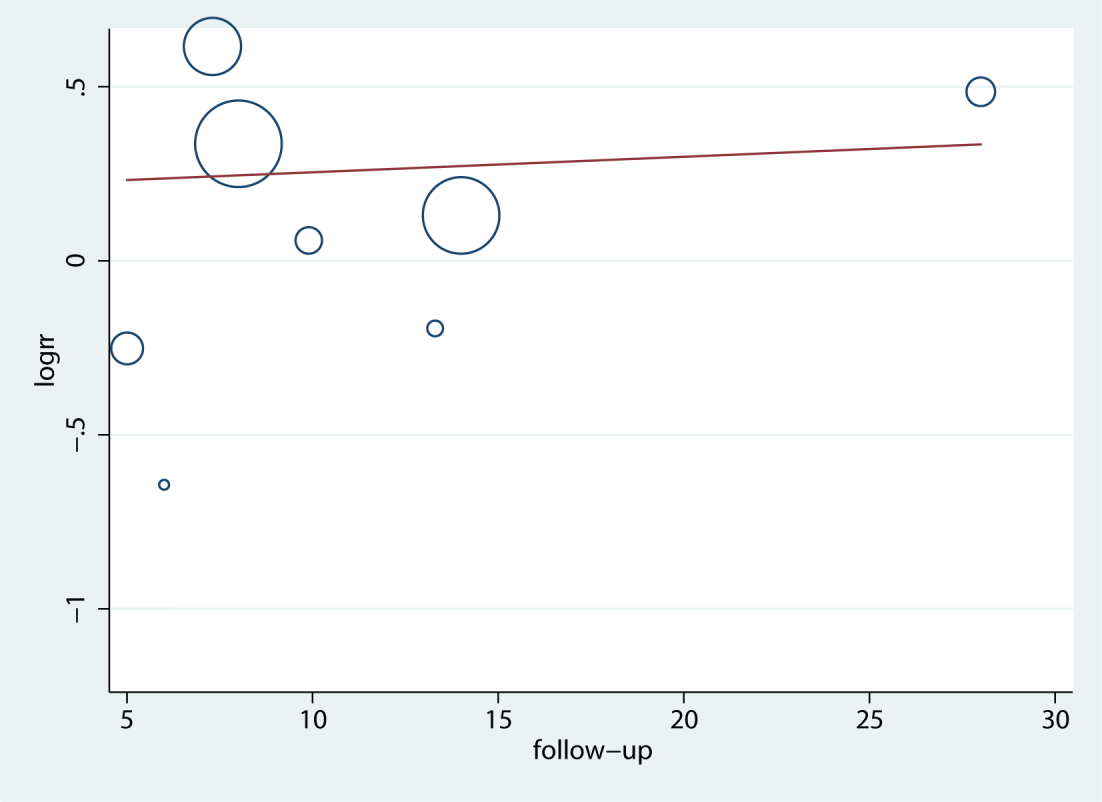


Figure S2. Meta-regression of sex differences in the relationship between former smokers and the risk of gastric cancer based on follow-up duration (P=0.840).
